# Genetic variability and functional implication of the long control region in HPV-16 variants in Southwest China

**DOI:** 10.1371/journal.pone.0182388

**Published:** 2017-08-02

**Authors:** Juemin Xi, Junying Chen, Miaoling Xu, Hongying Yang, Jia Luo, Yue Pan, Xiaodan Wang, Lijuan Qiu, Jiajia Yang, Qiangming Sun

**Affiliations:** 1 Institute of Medical Biology, Chinese Academy of Medical Sciences, and Peking Union Medical College, Kunming, People's Republic of China; 2 Yunnan Key Laboratory of Vaccine Research & Development on Severe Infectious Diseases, Kunming, People's Republic of China; 3 Yunnan Key Laboratory of Vector-borne Infectious Disease, Kunming, People's Republic of China; 4 The First Affiliated Hospital of Kunming Medical University, Kunming, People's Republic of China; 5 The Third Affiliated Hospital of Kunming Medical University, Yunnan Provincial Tumor Hospital, Kunming, People's Republic of China; 6 Kunming Medical University, Kunming, People's Republic of China; Fondazione IRCCS Istituto Nazionale dei Tumori, ITALY

## Abstract

HPV-16 long control region (LCR) has been shown to be the most variable region of the HPV-16 genome and may play important roles in viral persistence and the development of cervical cancer. This study aimed to assess the risk of HPV-16 LCR variants for cervical cancer in women of Southwest China. 2146 cervical scrapings of volunteer outpatients and 74 cervical cancer tissues were screened.14 entire HPV-16 LCRs from asymptomatic carriers and 34 entire HPV-16 LCRs from cervical cancer patients were successfully amplified and sequenced to align to others described. 58 different point mutations were detected in 54 nucleotide sites of HPV-16 LCR. G7193T and G7521A variants, accounting for 100% of the infections, were predicted to locate at the binding site for FOXA1 and SOX9, respectively. A7730C variant which showed a high mutation frequency in cervical cancer was predicted to be a binding site for the cellular transcription factor PHOX2A. In addition, phylogenetic analysis displayed a high prevalence of A lineage in HPV-16 LCR in this Southwest China population. This study may help understanding of the intrinsic geographical relatedness and the correlations between LCR mutations and the development of carcinogenic lesions in Southwest China population. And it provides useful data for the further study of the biological function of HPV-16 LCR variants.

## Introduction

Human papillomavirus type 16 (HPV-16) is prevalent worldwide and is the main etiological agent of cervical cancer [1]. HPV-16 variants have been grouped into the four major variant lineages and nine sublineages: (1) lineage A, including A1, A2, A3 (previously known as European) and A4 (Asian) sublineages; (2) lineage B, including B1 (Afr1a) and B2 (Afr1b) sublineages; (3) lineage C (African-2); and (4) lineage D, including D1 (North American, NA1), D2 (Asian-American, AA2), and D3 (Asian-American, AA1) sublineages [2].

HPV-16 is a capsid-enclosed circular double-stranded DNA virus from the papillomavirus family with a genome approximately 7.9 kb in length, consisting of an early region (E6, E7, E1, E2, E4, E5), a late region (L2, L1), a small non-coding region (NCR) localized between E5 and L2, and a long control region (LCR) [3], which is approximately 850 bp in length and is the most variable region of HPV-16.

The LCR region contains the early promoter and various transcriptional regulatory sites for both viral and cellular proteins [4–6], such as E2, YY1, Oct-1, NF1, transcriptional enhancer factor-1 (TEF-1), and others. Based principally on the LCR sequences, several studies from the United States, Germany, and Europe have suggested that certain LCR variants of HPV-16 may play an important role in viral persistence and the development of cervical cancer. For example, the E2 protein has been shown to be primarily a transcriptional repressor, and mutations of the E2 binding sites in LCR reduced P97 E2-mediated repression [7,8]. A study by Dong et al. implied that deletions or mutations of YY1-binding sites played a significant role in the over-expression of viral oncogenes and tumor progression [9,10]. Thus far, little has been reported on the HPV-16 LCR variants in China.

In this study, samples from asymptomatic carriers and cervical cancer patients were obtained from women in Southwest China, and polymorphic sites within the HPV-16 LCR were detected and compared to investigate the association of specific LCR mutations with the development of cervical cancer. A phylogenetic study and functional prediction of HPV-16 LCR in asymptomatic and cervical cancer specimens were also performed to determine the circulating lineages and investigate the polymorphisms of transcription factor binding sites of the HPV-16 LCR variants in Southwest China.

## Materials and methods

### Ethical statement

All participants were informed of the study aims, and a written informed consent was received from each patient before sample collection. The study was conducted with the approval of the Institutional Ethics Committee of Institute of Medical Biology, Chinese Academy of Medical Sciences & Peking Union Medical College and was performed in accordance with the Declaration of Helsinki for Human Research of 1974 (last modified in 2000).

### Collection of clinical specimens

Cervical scrapings of 2,146 volunteer outpatients were collected from the First Affiliated Hospital of Kunming Medical University in 2015. 74 cervical cancer samples were drawn from Yunnan Provincial Tumor Hospital of Kunming Medical University. After routine cytology and HC2 testing, each sample was transferred to a 1.5 ml Eppendorf tube.

### DNA extraction and HPV genotyping

The extraction of specimen DNA of frozen scraped cells was performed as previously described [11]. Briefly, the cervical scraping cell suspension was first centrifuged at 13,000 rpm for 5 min. The pellet was washed in 1 ml washing buffer (50 mM Tris-HCl, 1 mM EDTA, 0.5% Tween 20, pH 8.1) and then resuspended in a 100 μl digestion buffer (50 mM Tris-HCl, 0.1 mg/ml proteinase K, 1 mM EDTA, 0.5% Tween 20) at 55°C overnight. The digested pellet was boiled at 95°C for 10 min, followed by centrifugation at 13,000 rpm for 5 min. The supernatant was collected and stored at -80°C for later amplification of HPV DNA.

HPV genotyping was performed using a nested PCR assay. The extracted DNA was first amplified by the MY09/MY11 primer pair (MY09: CGTCCAAAAGGAAACTGAGC, MY11: GCACAGGGACATAACAATGG) followed by GP5+/GP6+ primer pair (GP5+: TTTGTTACTGTGGTAGATACTAC, GP6+: GAAAAATAAACTGTAAATCATATTC) according to the previous publications [11–13]. The first-round PCR was performed in a 20 μl total volume containing 1 μl extracted DNA template, 10 μl 2× Power Taq PCR MasterMix (BioTeke), 2 μl 20mM Primer MY09, 2 μl 20mM Primer MY11 and 5 μl H_2_O with the following parameters for the reactions: an initial heating at 94°C for 5 min, followed by 38 cycles of 94°C for 1 min, 55°C for 1 min, 72°C for 1 min, and a final elongation at 72°C for 10 min. In the second-round PCR, 5 μl of the first-round products along with 25 μl 2× Power Taq PCR MasterMix (BioTeke), 2 μl 20mM Primer GP5+, 2 μl 20mM Primer GP6+ and 5 μl H_2_O were brought to a volume of 50 μl and subjected to thermocycling as follows: 95°C for 5 min, followed by 38 cycles of 95°C for 1 min, 50°C for 1 min, 72°C for 50 s, and then 72°C for 10 min. β-globin (For: ACACAACTGTGTTCACTAGC, Rev: CAACTTCATCCACGTTCACC) was used as a positive control [14], and the PCR reaction mixture without the template DNA was served as a negative control. The PCR products were visualized on 1% agarose gels stained with GoldView^™^ nucleic acid stain. Positive samples with a single 150 bp band were then subjected to DNA sequencing and aligning on NCBI. Then the score, query cover, E value and identity of each alignment were taken into overall consideration for HPV genotyping.

### Amplification and sequencing of HPV-16 LCR

According to the HPV reference strain (NCBI Reference Sequence: NC_001526.3), two pairs of primers that were used in the amplification of the two partially overlapping fragments were designed to cover the entire HPV-16 LCR as listed in Table 1. All primers were synthesized by Sangon Biotech (Shanghai, China). The amplification of the LCR fragments was performed in 50 μl reaction volumes containing 2 μl of extracted DNA and 2× Power Taq PCR MasterMix (BioTeke). The following conditions were used for the PCR: 95°C for 5 min, followed by 40 cycles of 95°C for 30 s, 57°C for 45 s, 72°C for 50 s, and then 72°C for 10 min. The PCR products were examined under UV light after electrophoretic separation on a 1% agarose gel. The positive DNA fragments were directed to bidirectional DNA sequencing (TSINGKE, China) for further analysis. The obtained HPV-16 LCR sequences in this study were deposited in the NCBI GenBank database (https://www.ncbi.nlm.nih.gov/genbank/) under the accession number: KX912865 to KX912878 for the sequences from asymptomatic carriers, and KX912879 to KX912912 for the sequences from cervical cancer patients.

**Table 1:** Primer pairs designed for complete LCR amplification of HPV-16.

Primer name	Sequence	Position	Amplicon size (bp)
**LCR 1F**	ACCCACCACCTCATCTACCTCTACAA	7101–7126	460
**LCR 1R**	ATTTGGCACGCATGGCAAGCAGGAA	7560–7536	
**LCR 2F**	CATGCTTTTTGGCACAAAATGTGTTTT	7465–7491	621
**LCR 2R**	ATATCATGTATAGTTGTTTGCAGCTCT	180–154	

### Variant identification and phylogenetic analysis

Molecular characterization was performed by a sequence analysis of HPV-16 LCR amplicons. To identify mutational sites in LCR, all of the studied LCR sequences were aligned to the HPV-16 reference sequence (NC_001526.3) by MEGA 7.0 package [15] after direct sequencing.

The phylogenetic tree of HPV-16 LCR was constructed by the Neighbor-Joining method and the Kimura 2-Parameter model by MEGA package 7.0. The number of bootstrap replications was 1,000. The reference HPV-16 LCR sequences that were used to construct the phylogenetic branches were collected from the GenBank sequence database and included NC_001526.3, K02718 (A1), AF536179 (A2), HQ644236 (A3), AF534061 (A4), AF536180 (B1), HQ644298 (B2), AF472509 (C), HQ644257 (D1), HQ644270 (D2) and AF402678 (D3).

### Prediction of transcription factor binding sites

The high-quality transcription factor binding profile database JASPAR (http://jaspar.genereg.net/) [16,17] was used to investigate the potential binding sites within HPV-16 LCR, including sites for YY1, CEBPB, SP1, SRY, FOXA, SOX9, PHOX2A, JUN, FOS, FOXA1, HSF1, ETS1, NFIL3, IRF1, IRF2, SPIB, REL, MAFK, STAT1, STAT3, HOXC11, SRF, RAX, VAX1, IRF7, NFKB1. The relative profile score threshold was set at 85%.

### Statistical analysis

To examine the distributions of the HPV 16 LCR mutations with respect to tumorigenicity of cervical cancer, a Fisher’s exact test was performed. A P-value less than 0.05 was considered statistically significant. The entire database was analyzed with SPSS 12.0.

## Results

### HPV prevalence in Southwest China

**Fig 1:**
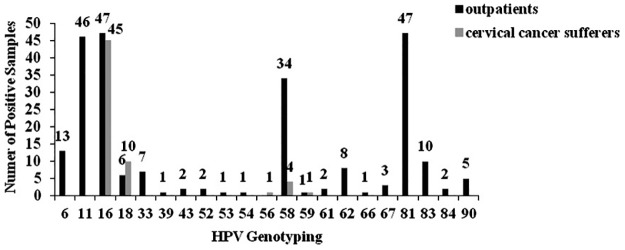
HPV genotype distribution of outpatients and cervical cancer sufferers. HPV genotyping was performed using a nested PCR assay. The distribution of HPV genotype among HPV-positive outpatients and cervical cancer sufferers were shown.

Of the 2146 collected outpatient samples, 248 samples were HPV-positive. By genotyping, 21 HPV types were identified, including HPV-6, 11, 16, 18, 33, 39, 43, 52, 53, 54, 58, 59, 61, 62, 66, 67, 81, 83, 84, 90 and 91. Of the types observed, HPV-16, 81, 11, 58, and 6 were the five most common types, accounting for 72.58% (180/248). The most common high-risk and low-risk types were HPV-16 (18.95%, 47/248) and HPV-81 (18.95%, 47/248), respectively (Fig 1A and 1B).

Of the 74 cervical cancer samples, 61 samples were identified HPV positive and belonging to high-risk types. They were HPV-16, 18, 58, 56 and 59, accounting for 73.77% (45/61), 16.39% (10/61), 6.56% (4/61), 1.64% (1/61), 1.64% (1/61), respectively.

### Genomic polymorphisms of HPV-16 LCR

Among the 47 HPV-16-positive samples from the outpatients (asymptomatic carriers), only 14 entire LCR sequences were obtained. A possible explanation is the small copy number of HPV in some women. Meanwhile, 34 entire LCR sequences were obtained from the cervical cancer samples. When aligned to the reference LCR, nucleotide variations of HPV-16 LCR were found in all of the studied HPV-16 positive asymptomatic carrier samples (14/14) and cervical cancer samples (34/34). In total, 58 different point mutations were detected in 54 nucleotide sites within the LCR segment with 832 bp in length, and mutations occurred over a span of nucleotides 7157 to 82 (Table 2 and S1 Table). The mutation frequency varied from 2% (1/48) to 100% (48/48), among which the G7193T and G7521A variants got a mutation frequency of 100%. The other common LCR changes were A7730C, A7175C, T7177C, T7201C, C7270T, G7842A, C24T, A7287C and A7289C with mutation frequencies of approximately 71% (34/48), 65% (31/48), 65% (31/48), 65% (31/48), 65% (31/48), 65% (31/48), 65% (31/48), 63% (30/48) and 46% (22/48), respectively. No insertion or deletion mutation sites were found.

**Table 2:** Nucleotide variations of HPV-16 LCR and the proposed binding sites for transcription factors.

Nucleotide mutation site	Asymptomatic carriers (n = 14)		Cervical cancers (n = 34)		Total (N = 48)		Transcription factors
	Number of cases	Proportion (%)	Number of cases	Proportion (%)	Number of cases	Proportion (%)	
**A7168G**	2	14	5	15	7	15	FOXA1
**A7174C**	1	7	0	0	1	2	FOXA1
**A7175C**	7	50	24	71	31	65	FOXA1
**T7176G**	1	7	0	0	1	2	FOXA1
**T7177C**	7	50	24	71	31	65	FOXA1
**T7177A**	1	7	0	0	1	2	FOXA1
**G7193T**	14	100	34	100	48	100	FOXA1
**G7219C**	0	0	1	3	1	2	STAT3, HOXC11
**C7270T**	7	50	24	71	31	65	FOXA1
**C7310T**	0	0	2	6	2	4	CEBPB, SRY
**T7328C**	1	7	0	0	1	2	SOX9
**T7393A**	1	7	0	0	1	2	ETS1
**C7394T**	0	0	2	6	2	4	ETS1
**C7395T**	0	0	4	12	4	8	ETS1
**C7505T**	2	14	0	0	2	4	MAFK
**A7507T**	0	0	1	3	1	2	MAFK
**G7521A**	14	100	34	100	48	100	SOX9
**A7636C**	1	7	0	0	1	3	FOS
**A7660G**	1	7	0	0	1	3	HOXC11
**A7729C**	0	0	2	6	2	4	PHOX2A
**A7730C**	7	50	27	79	34	71	PHOX2A
**T7743G**	0	0	2	6	2	4	PHOX2A, RAX
**G7826A**	0	0	2	6	2	4	SOX9, SRY
**A7830C**	0	0	2	6	2	4	SRY
**C7886G**	0	0	2	6	2	4	STAT3
**A7900C**	1	7	0	0	1	2	NFIL3, SRY

When compared with the asymptomatic carriers, A7175C, T7177C, T7201C, C7270T, A7287C, A7289C, A7730C, G7842A and C24T got higher mutation frequencies in cervical cancer. Additionally, the variation sites such as A7233C and C7395T were only detected in cervical cancer (Table 2 and S1 Table). These may suggest a possible relationship between these variation sites and cervical cancers.

### Phylogenetic analysis of HPV-16 LCR

**Fig 2:**
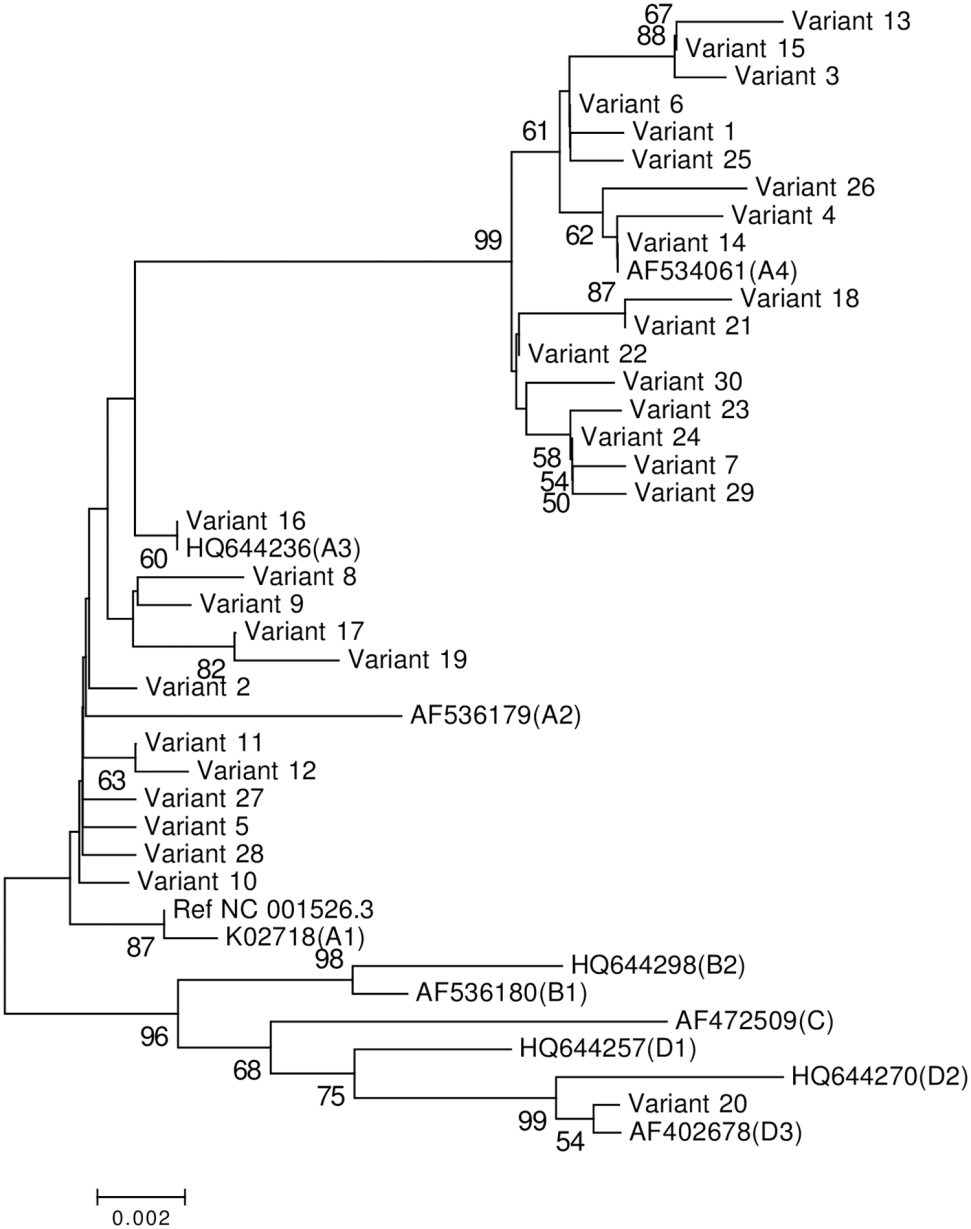
Phylogenetic tree of the HPV-16 variants based on LCR sequences. The Neighbor-Joining method and the Kimura 2-Parameter model were used to construct the phylogenetic tree by MEGA package 7.0. The standard sequences included NC_001526.3, K02718 (A1), AF536179 (A2), HQ644236 (A3), AF534061 (A4), AF536180 (B1), HQ644298 (B2), AF472509 (C), HQ644257 (D1), HQ644270 (D2) and AF402678 (D3). The lineage to which each accession number belongs was shown in a parenthesis. Numbers closest to the branch points are bootstrap values (1,000 replicates). Values lower than 50% are not shown.

When considering the entire LCR sequence, combinations of the single nucleotide polymorphisms (SNPs) resulted in 30 unique sequences (variant IDs: 1 to 30) (S1 Table). Variant IDs 1, 5, 6, 14, 15, 16, 20, 22, 26 represented 6, 2, 2, 4, 4, 3, 2, 2 and 2 samples, respectively. As shown in Fig 2 a Neighbor-Joining tree of HPV-16 LCR based on the Kimura 2-Parameter model was constructed from a molecular phylogenetic analysis of 11 reference LCR nucleotide sequences aligned with 30 unique variants with 1,000 bootstrap replicates to test the robustness of the phylogenetic groups. A total of 29 vairants (representing 32 cervical cancer samples and 14 asymptomatic samples), accounting for 97% (29/30), were clustered in A lineage, including 17 A4 (representing 24 cervical cancer samples and 7 asymptomatic samples) and 12 A2-3 (representing 8 cervical cancer samples and 7 asymptomatic samples) with the proportions of 59% (17/29) and 41% (12/29), respectively. Meanwhile, 1 vairant (representing 2 cervical cancer samples),fell into D lineage, accounting for 3% (1/30).

### Variants in the transcription factor binding sites

**Fig 3:**
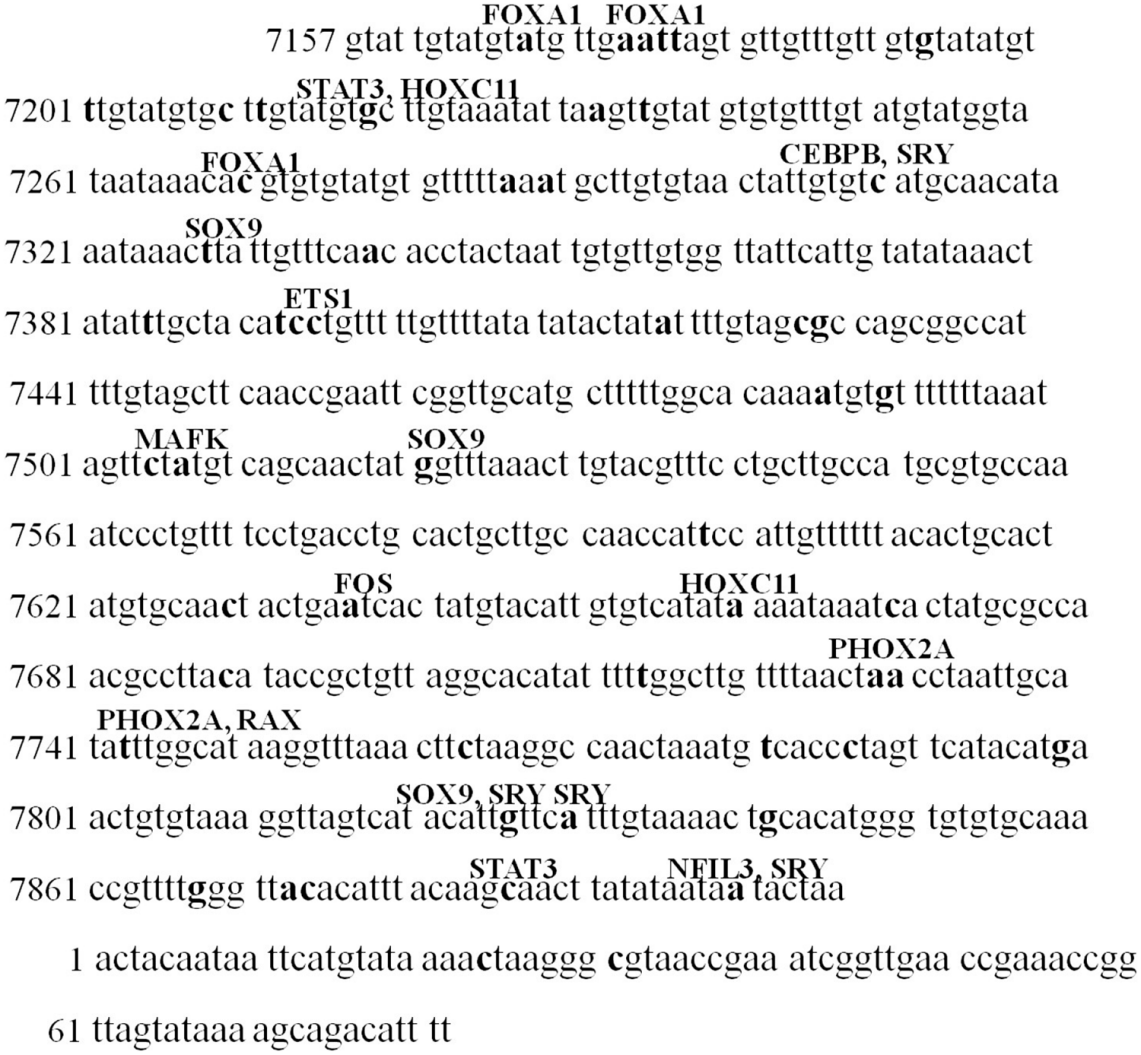
The potential binding sites of cellular transcription factors within HPV-16 LCR. The JASPAR database was used to investigate the potential binding sites within HPV-16 LCR. The proposed binding sites for transcription factors were separately marked on the bold nucleotide mutation sites.

To assess the effects of the variations in HPV-16 LCR on binding sites of cellular transcription factors, the JASPAR database was used. The results showed that several variations in the HPV-16 LCR spanning nucleotides 7157 to 7906 altered the binding sites of transcription factors (Fig 3 and Table 2), which potentially influenced the binding of transcription factors and resultant gene expression.

G7193T and G7521A variants, which accounted for 100% of the infections, were located at the binding site for Forkhead box protein A1 (FOXA1) and sex-determining region Y-box 9 (SOX9), respectively. The variations of A7730C contributed to influence the binding of paired-like homeobox 2a (PHOX2A). Furthermore, the variations of C7310T, C7395T and C7886G were predicted to locate at the binding site for CCAAT/enhancer binding protein beta (CEBPB), ETS proto-oncogene 1 (ETS1) and signal transducer and activator of transcription 3 (STAT3), respectively. While, the mutations C24T and C31T within 1 to 82 nucleotides of the HPV-16 LCR were not predicted to affect the binding sites of transcription factors.

## Discussion

More than 170 different types have been characterized in the HPV family [18]. HPV preferentially infects the mucosa of the genitals, upper respiratory tract, and skin. Compared with our previous study on HPV prevalence in Southwest China from 2009 to 2011 [19], HPV-39, 52, 53, 54, 61, 62, 67, 84, 90 and 91 were newly detected in this study. HPV-16 was still the most prevalent high-risk type in Southwest China. Among the 248 HPV-positive asymptomatic carrier samples detected in this study, 47 cases (18.95%) were HPV-16 positive.

HPV-16 variants have been found to show different geographic distributions [2] and exhibit diverse oncogenic potentials. Several studies have indicated that specific lineages may have effects on the persistence of HPV infection and the progression of cervical cancer precursor lesions [20–24]. In HPV-16 LCR variants of a Costa Rican patient population, non-European variants were detected in 5.8% of control subjects, while they were detected in 43.7% of cancers and 14.3% high-grade squamous intraepithelial lesions (HSILs) [22]. In this study, more than half of asymptomatic carriers fell into A4 (previously known as Asian) phylogenetic branches, which may indicate an even higher risk for disease progression. Also, a high prevalence of A lineage in the HPV-16 LCR in Southwest China indicates a possible epidemiological linkage among Asia, Europe and China.

The LCR has been shown to be the most variable region of the HPV-16 genome in different populations [10] and contains most of the regulatory elements. Much more phylogenetic information has been confirmed within the LCR than within oncogene E6 [4]. Certain changes within the LCR region can lead to the addition or loss of binding sites for transcription factors. Through the effects on the formation of regulatory protein complexes with viral DNA, nucleotide changes within the LCR region can influence the transcription of viral genes [10], especially oncogenes E6 and E7. A transcriptional analysis of the HPV-16 LCR is essential to dissect the association between LCR nucleotide variations and carcinogenicity of HPV-16 variants. In this study, 58 different point mutations were detected at 54 nucleotide sites. The presence of A7730C variants in LCR, which was further shown to be a potential binding site for PHOX2A, a transcription factor involving in cell proliferation and migration in lung cancer [25], showed a high mutation frequency in cervical cancer. Therefore, it may be a warning for the progression and promotion of cervical cancer development. Further study should be performed to demonstrate its biological function. In addition, the nucleotide changes G7193T and G7521A, which accounted for 100% of the infections in our study, were the most prevalent. The variation of G7193T was predicted at the binding site for the cellular transcription factor FOXA1, which has been reported as a pioneer transcription factor that regulates the progression of cancer in the breast, liver, prostate, lung, and endometrium [26–30]. The G7521A variant was located at the binding site of the transcriptional repressor SOX9, a potential tumor suppressor in cervical cancer, and suppressesing cervical tumor growth through transactivating p21WAF1/CIP1 [31].

Compared to the control group of HPV16-positive women without HSIL or cervical cancer, those polymorphic sites only found within the HPV-16 LCR region of isolates from cervical cancer patients, should receive attentions. The variations of C7394T and C7395T were predicted within the binding site for Ets transcription factor 1, which has been demonstrated to play roles in the complex biological control of tumor progression [32, 33]. CEBPB, which potentially bond at the site of 7310, was identified to take a part in cervical carcinogenesis [34]. Whether these variants participate in malignancy of HPV-16-infected cells is still investigated.

## Conclusions

In this study, 58 different point mutations were detected in 54 nucleotide sites of HPV-16 LCR. Phylogenetic analysis displayed a high prevalence of A lineage in HPV-16 LCR in this Southwest China population. G7193T and G7521A detected in all the infections, were predicted to locate at the binding site for FOXA1 and SOX9, respectively. In addition, A7730C variant, which showed a high mutation frequency in cervical cancer, was predicted to be a binding site for the cellular transcription factor PHOX2A. Our study provided a helpful experimental basis for understanding the intrinsic geographical relatedness of HPV-16 variants, the interaction of HPV-16 LCR mutation variants and transcription factors, and the correlation between mutations of the LCR region and carcinogenesis. It also helps performing further study to demonstrate the biological function of HPV-16 LCR variants.

## Supporting information

S1 Table HPV-16 variants based on the LCR sequences. (DOC)
