# Supplementary material for: Genetic variability and functional implication of the long control region in HPV-16 variants in Southwest China
Source: PLoS One. 2017 Aug 2;12(8):e0182388. doi: 10.1371/journal.pone.0182388 (PMC5540483; doi:10.1371/journal.pone.0182388)
Supplement: S1 Table — (DOC) [file pone.0182388.s001.doc]

**Supporting information**

**S1 Table. HPV-16 variants based on the LCR sequences**

| **GenBank accession** | **Variant ID** | **Nucleotide mutation site** | | | | | | | | | | | | | | | | | |
| --- | --- | --- | --- | --- | --- | --- | --- | --- | --- | --- | --- | --- | --- | --- | --- | --- | --- | --- | --- |
|  |  | **7168** | **7174** | **7175** | **7176** | **7177** | **7193** | **7201** | **7210** | **7212** | **7219** | **7233** | **7236** | **7270** | **7287** | **7289** | **7310** | **7328** | **7339** |
| **NC_001526.3** |  | A | A | A | T | T | G | T | C | T | G | A | T | C | A | A | C | T | A |
| **KX912865** | 1 | - | - | C | - | C | T | C | - | - | - | - | - | T | C | C | - | - | - |
| **KX912876** | 1 | - | - | C | - | C | T | C | - | - | - | - | - | T | C | C | - | - | - |
| **KX912889** | 1 | - | - | C | - | C | T | C | - | - | - | - | - | T | C | C | - | - | - |
| **KX912890** | 1 | - | - | C | - | C | T | C | - | - | - | - | - | T | C | C | - | - | - |
| **KX912891** | 1 | - | - | C | - | C | T | C | - | - | - | - | - | T | C | C | - | - | - |
| **KX912892** | 1 | - | - | C | - | C | T | C | - | - | - | - | - | T | C | C | - | - | - |
| **KX912866** | 2 | - | - | - | - | A | T | - | - | - | - | - | - | - | - | - | - | - | - |
| **KX912867** | 3 | - | - | C | - | C | T | C | - | - | - | - | - | T | C | C | - | - | - |
| **KX912868** | 4 | - | - | C | - | C | T | C | A | - | - | G | - | T | C | C | - | - | - |
| **KX912869** | 5 | - | - | - | - | - | T | - | - | - | - | - | - | - | - | - | - | - | - |
| **KX912893** | 5 | - | - | - | - | - | T | - | - | - | - | - | - | - | - | - | - | - | - |
| **KX912870** | 6 | - | - | C | - | C | T | C | - | - | - | - | - | T | C | C | - | - | - |
| **KX912897** | 6 | - | - | C | - | C | T | C | - | - | - | - | - | T | C | C | - | - | - |
| **KX912871** | 7 | - | - | C | - | C | T | C | - | - | - | - | - | T | C | - | - | - | - |
| **KX912872** | 8 | G | C | - | - | - | T | - | - | - | - | - | - | - | - | - | - | - | - |
| **KX912873** | 9 | G | - | - | G | - | T | - | - | - | - | - | - | - | - | - | - | - | - |
| **KX912874** | 10 | - | - | - | - | - | T | - | - | - | - | - | - | - | - | - | - | C | - |
| **KX912875** | 11 | - | - | - | - | - | T | - | - | - | - | - | - | - | - | - | - | - | - |
| **KX912877** | 12 | - | - | - | - | - | T | - | - | - | - | - | - | - | - | - | - | - | - |
| **KX912878** | 13 | - | - | C | - | C | T | C | - | - | - | - | - | T | C | C | - | - | - |
| **KX912879** | 14 | - | - | C | - | C | T | C | - | - | - | - | - | T | C | C | - | - | - |
| **KX912898** | 14 | - | - | C | - | C | T | C | - | - | - | - | - | T | C | C | - | - | - |
| **KX912904** | 14 | - | - | C | - | C | T | C | - | - | - | - | - | T | C | C | - | - | - |
| **KX912905** | 14 | - | - | C | - | C | T | C | - | - | - | - | - | T | C | C | - | - | - |
| **KX912880** | 15 | - | - | C | - | C | T | C | - | - | - | - | - | T | C | C | - | - | - |
| **KX912881** | 15 | - | - | C | - | C | T | C | - | - | - | - | - | T | C | C | - | - | - |
| **KX912903** | 15 | - | - | C | - | C | T | C | - | - | - | - | - | T | C | C | - | - | - |
| **KX912909** | 15 | - | - | C | - | C | T | C | - | - | - | - | - | T | C | C | - | - | - |
| **KX912882** | 16 | G | - | - | - | - | T | - | - | - | - | - | - | - | - | - | - | - | - |
| **KX912895** | 16 | G | - | - | - | - | T | - | - | - | - | - | - | - | - | - | - | - | - |
| **KX912912** | 16 | G | - | - | - | - | T | - | - | - | - | - | - | - | - | - | - | - | - |
| **KX912883** | 17 | G | - | - | - | - | T | - | - | - | - | - | - | - | - | - | T | - | - |
| **KX912884** | 18 | - | - | C | - | C | T | C | - | G | - | - | C | T | C | - | - | - | - |
| **KX912885** | 19 | G | - | - | - | - | T | - | - | - | - | C | - | - | - | - | T | - | - |
| **KX912886** | 20 | - | - | - | - | - | T | - | - | - | - | C | - | - | - | - | - | - | T |
| **KX912887** | 20 | - | - | - | - | - | T | - | - | - | - | C | - | - | - | - | - | - | T |
| **KX912888** | 21 | - | - | C | - | C | T | C | - | - | - | - | - | T | C | - | - | - | - |
| **KX912894** | 22 | - | - | C | - | C | T | C | - | - | - | - | - | T | C | - | - | - | - |
| **KX912896** | 22 | - | - | C | - | C | T | C | - | - | - | - | - | T | C | - | - | - | - |
| **KX912899** | 23 | - | - | C | - | C | T | C | - | - | - | - | - | T | C | - | - | - | - |
| **KX912900** | 24 | - | - | C | - | C | T | C | - | - | - | - | - | T | C | - | - | - | - |
| **KX912901** | 25 | - | - | C | - | C | T | C | - | - | - | - | - | T | C | C | - | - | - |
| **KX912911** | 25 | - | - | C | - | C | T | C | - | - | - | - | - | T | C | C | - | - | - |
| **KX912902** | 26 | - | - | C | - | C | T | C | - | - | C | - | - | T | T | C | - | - | - |
| **KX912906** | 27 | - | - | - | - | - | T | - | - | - | - | - | - | - | - | - | - | - | - |
| **KX912907** | 28 | - | - | - | - | - | T | - | - | - | - | - | - | - | - | - | - | - | - |
| **KX912908** | 29 | - | - | C | - | C | T | C | - | - | - | - | - | T | C | - | - | - | - |
| **KX912910** | 30 | - | - | C | - | C | T | C | - | - | - | - | - | T | C | - | - | - | - |

**S1 Table. HPV-16 variants based on the LCR sequences (Continued Ⅰ)**

| **GenBank accession** | **Variant ID** | **Nucleotide mutation site** | | | | | | | | | | | | | | | | | |
| --- | --- | --- | --- | --- | --- | --- | --- | --- | --- | --- | --- | --- | --- | --- | --- | --- | --- | --- | --- |
|  |  | **7385** | **7393** | **7394** | **7395** | **7419** | **7428** | **7429** | **7485** | **7489** | **7505** | **7507** | **7521** | **7598** | **7629** | **7636** | **7660** | **7669** | **7689** |
| **NC_001526.3** |  | T | T | C | C | A | C | G | A | G | C | A | G | T | C | A | A | C | C |
| **KX912865** | 1 | - | - | - | - | G | - | - | - | - | - | - | A | - | - | - | - | - | - |
| **KX912876** | 1 | - | - | - | - | G | - | - | - | - | - | - | A | - | - | - | - | - | - |
| **KX912889** | 1 | - | - | - | - | G | - | - | - | - | - | - | A | - | - | - | - | - | - |
| **KX912890** | 1 | - | - | - | - | G | - | - | - | - | - | - | A | - | - | - | - | - | - |
| **KX912891** | 1 | - | - | - | - | G | - | - | - | - | - | - | A | - | - | - | - | - | - |
| **KX912892** | 1 | - | - | - | - | G | - | - | - | - | - | - | A | - | - | - | - | - | - |
| **KX912866** | 2 | - | - | - | - | - | - | - | - | - | - | - | A | - | - | - | - | - | - |
| **KX912867** | 3 | - | - | - | - | - | - | A | - | - | T | - | A | - | - | - | - | - | - |
| **KX912868** | 4 | - | - | - | - | - | - | - | - | - | - | - | A | - | - | - | - | - | - |
| **KX912869** | 5 | - | - | - | - | - | - | - | - | - | - | - | A | - | - | - | - | - | - |
| **KX912893** | 5 | - | - | - | - | - | - | - | - | - | - | - | A | - | - | - | - | - | - |
| **KX912870** | 6 | - | - | - | - | - | - | - | - | - | - | - | A | - | - | - | - | - | - |
| **KX912897** | 6 | - | - | - | - | - | - | - | - | - | - | - | A | - | - | - | - | - | - |
| **KX912871** | 7 | - | A | - | - | - | - | - | - | - | - | - | A | - | - | - | G | - | - |
| **KX912872** | 8 | - | - | - | - | - | - | - | - | - | T | - | A | - | - | - | - | - | - |
| **KX912873** | 9 | - | - | - | - | - | - | - | - | - | - | - | A | - | - | - | - | - | - |
| **KX912874** | 10 | - | - | - | - | - | - | - | - | - | - | - | A | - | - | - | - | - | - |
| **KX912875** | 11 | - | - | - | - | - | - | - | - | - | - | - | A | - | - | - | - | - | - |
| **KX912877** | 12 | - | - | - | - | - | - | - | - | - | - | - | A | - | - | - | - | - | - |
| **KX912878** | 13 | - | - | - | - | - | - | A | - | - | - | - | A | - | - | C | - | - | - |
| **KX912879** | 14 | - | - | - | - | - | - | - | - | - | - | - | A | - | - | - | - | - | - |
| **KX912898** | 14 | - | - | - | - | - | - | - | - | - | - | - | A | - | - | - | - | - | - |
| **KX912904** | 14 | - | - | - | - | - | - | - | - | - | - | - | A | - | - | - | - | - | - |
| **KX912905** | 14 | - | - | - | - | - | - | - | - | - | - | - | A | - | - | - | - | - | - |
| **KX912880** | 15 | - | - | - | - | - | - | A | - | - | - | - | A | - | - | - | - | - | - |
| **KX912881** | 15 | - | - | - | - | - | - | A | - | - | - | - | A | - | - | - | - | - | - |
| **KX912903** | 15 | - | - | - | - | - | - | A | - | - | - | - | A | - | - | - | - | - | - |
| **KX912909** | 15 | - | - | - | - | - | - | A | - | - | - | - | A | - | - | - | - | - | - |
| **KX912882** | 16 | - | - | - | - | - | - | - | - | - | - | - | A | - | - | - | - | - | - |
| **KX912895** | 16 | - | - | - | - | - | - | - | - | - | - | - | A | - | - | - | - | - | - |
| **KX912912** | 16 | - | - | - | - | - | - | - | - | - | - | - | A | - | - | - | - | - | - |
| **KX912883** | 17 | - | - | - | T | - | - | - | - | - | - | - | A | - | - | - | - | - | - |
| **KX912884** | 18 | - | - | - | - | - | - | - | - | - | - | - | A | - | - | - | - | - | - |
| **KX912885** | 19 | - | - | - | T | - | - | - | - | - | - | T | A | - | - | - | - | - | - |
| **KX912886** | 20 | - | - | T | T | - | - | - | C | A | - | - | A | - | - | - | - | T | A |
| **KX912887** | 20 | - | - | T | T | - | - | - | C | A | - | - | A | - | - | - | - | T | A |
| **KX912888** | 21 | - | - | - | - | - | - | - | - | - | - | - | A | - | - | - | - | - | - |
| **KX912894** | 22 | - | - | - | - | - | - | - | - | - | - | - | A | - | - | - | - | - | - |
| **KX912896** | 22 | - | - | - | - | - | - | - | - | - | - | - | A | - | - | - | - | - | - |
| **KX912899** | 23 | - | - | - | - | - | - | - | - | - | - | - | A | - | - | - | G | - | - |
| **KX912900** | 24 | - | - | - | - | - | - | - | - | - | - | - | A | - | - | - | G | - | - |
| **KX912901** | 25 | - | - | - | - | - | - | - | - | - | - | - | A | - | - | - | - | - | - |
| **KX912911** | 25 | - | - | - | - | - | - | - | - | - | - | - | A | - | - | - | - | - | - |
| **KX912902** | 26 | - | - | - | - | - | A | - | - | - | - | - | A | - | - | - | - | - | - |
| **KX912906** | 27 | - | - | - | - | - | - | - | - | - | - | - | A | G | - | - | - | - | - |
| **KX912907** | 28 | G | - | - | - | - | - | - | - | - | - | - | A | - | - | - | - | - | - |
| **KX912908** | 29 | - | - | - | - | - | - | - | - | - | - | - | A | - | T | - | G | - | - |
| **KX912910** | 30 | - | - | - | - | - | - | - | - | - | - | - | A | - | - | - | - | - | - |

**S1 Table. HPV-16 variants based on the LCR sequences (Continued Ⅱ)**

| **GenBank accession** | **Variant ID** | **Nucleotide mutation site** | | | | | | | | | | | | | | | | | |
| --- | --- | --- | --- | --- | --- | --- | --- | --- | --- | --- | --- | --- | --- | --- | --- | --- | --- | --- | --- |
|  |  | **7714** | **7729** | **7730** | **7743** | **7764** | **7781** | **7786** | **7799** | **7826** | **7830** | **7842** | **7868** | **7873** | **7874** | **7886** | **7900** | **24** | **31** |
| **NC_001526.3** |  | T | A | A | T | C | T | C | G | G | A | G | G | A | C | C | A | C | C |
| **KX912865** | 1 | - | - | C | - | - | - | - | - | - | - | A | - | - | - | - | - | T | - |
| **KX912876** | 1 | - | - | C | - | - | - | - | - | - | - | A | - | - | - | - | - | T | - |
| **KX912889** | 1 | - | - | C | - | - | - | - | - | - | - | A | - | - | - | - | - | T | - |
| **KX912890** | 1 | - | - | C | - | - | - | - | - | - | - | A | - | - | - | - | - | T | - |
| **KX912891** | 1 | - | - | C | - | - | - | - | - | - | - | A | - | - | - | - | - | T | - |
| **KX912892** | 1 | - | - | C | - | - | - | - | - | - | - | A | - | - | - | - | - | T | - |
| **KX912866** | 2 | - | - | - | - | - | - | - | - | - | - | - | - | - | - | - | - | - | - |
| **KX912867** | 3 | - | - | C | - | - | - | - | - | - | - | A | - | - | G | - | - | T | - |
| **KX912868** | 4 | - | - | C | - | - | C | - | - | - | - | A | - | - | - | - | - | T | - |
| **KX912869** | 5 | - | - | - | - | - | - | - | - | - | - | - | A | - | - | - | - | - | - |
| **KX912893** | 5 | - | - | - | - | - | - | - | - | - | - | - | A | - | - | - | - | - | - |
| **KX912870** | 6 | - | - | C | - | - | - | - | - | - | - | A | - | - | - | - | - | T | - |
| **KX912897** | 6 | - | - | C | - | - | - | - | - | - | - | A | - | - | - | - | - | T | - |
| **KX912871** | 7 | - | - | C | - | - | - | - | - | - | - | A | - | - | - | - | - | T | - |
| **KX912872** | 8 | - | - | - | - | - | - | - | - | - | - | - | - | - | - | - | - | - | - |
| **KX912873** | 9 | - | - | - | - | - | - | - | - | - | - | - | - | - | - | - | - | - | - |
| **KX912874** | 10 | - | - | - | - | - | - | - | - | - | - | - | - | - | - | - | - | - | - |
| **KX912875** | 11 | G | - | - | - | - | - | - | - | - | - | - | - | - | - | - | - | - | - |
| **KX912877** | 12 | G | - | - | - | - | - | - | C | - | - | - | - | - | - | - | - | - | - |
| **KX912878** | 13 | - | - | C | - | - | - | - | - | - | - | A | - | - | G | - | C | T | - |
| **KX912879** | 14 | - | - | C | - | - | C | - | - | - | - | A | - | - | - | - | - | T | - |
| **KX912898** | 14 | - | - | C | - | - | C | - | - | - | - | A | - | - | - | - | - | T | - |
| **KX912904** | 14 | - | - | C | - | - | C | - | - | - | - | A | - | - | - | - | - | T | - |
| **KX912905** | 14 | - | - | C | - | - | C | - | - | - | - | A | - | - | - | - | - | T | - |
| **KX912880** | 15 | - | - | C | - | - | - | - | - | - | - | A | - | - | G | - | - | T | - |
| **KX912881** | 15 | - | - | C | - | - | - | - | - | - | - | A | - | - | G | - | - | T | - |
| **KX912903** | 15 | - | - | C | - | - | - | - | - | - | - | A | - | - | G | - | - | T | - |
| **KX912909** | 15 | - | - | C | - | - | - | - | - | - | - | A | - | - | G | - | - | T | - |
| **KX912882** | 16 | - | - | C | - | - | - | - | - | - | - | - | - | - | - | - | - | - | - |
| **KX912895** | 16 | - | - | C | - | - | - | - | - | - | - | - | - | - | - | - | - | - | - |
| **KX912912** | 16 | - | - | C | - | - | - | - | - | - | - | - | - | - | - | - | - | - | - |
| **KX912883** | 17 | - | - | - | - | - | - | - | - | - | - | - | - | - | - | - | - | - | - |
| **KX912884** | 18 | - | - | C | - | - | - | - | - | A | - | A | - | G | - | - | - | T | - |
| **KX912885** | 19 | - | - | - | - | - | - | - | - | - | - | - | - | - | - | - | - | - | - |
| **KX912886** | 20 | - | C | - | G | T | - | T | - | - | - | - | - | - | - | G | - | - | - |
| **KX912887** | 20 | - | C | - | G | T | - | T | - | - | - | - | - | - | - | G | - | - | - |
| **KX912888** | 21 | - | - | C | - | - | - | - | - | A | - | A | - | G | - | - | - | T | - |
| **KX912894** | 22 | - | - | C | - | - | - | - | - | - | - | A | - | - | - | - | - | T | - |
| **KX912896** | 22 | - | - | C | - | - | - | - | - | - | - | A | - | - | - | - | - | T | - |
| **KX912899** | 23 | - | - | C | - | - | - | - | A | - | - | A | - | - | - | - | - | T | - |
| **KX912900** | 24 | - | - | C | - | - | - | - | - | - | - | A | - | - | - | - | - | T | - |
| **KX912901** | 25 | - | - | C | - | - | - | - | - | - | C | A | - | - | - | - | - | T | - |
| **KX912911** | 25 | - | - | C | - | - | - | - | - | - | C | A | - | - | - | - | - | T | - |
| **KX912902** | 26 | - | - | C | - | - | C | - | - | - | - | A | - | - | - | - | - | T | - |
| **KX912906** | 27 | - | - | - | - | - | - | - | - | - | - | - | - | - | - | - | - | - | - |
| **KX912907** | 28 | - | - | - | - | - | - | - | - | - | - | - | - | - | - | - | - | - | - |
| **KX912908** | 29 | - | - | C | - | - | - | - | - | - | - | A | - | - | - | - | - | T | - |
| **KX912910** | 30 | - | - | C | - | - | - | - | C | - | - | A | - | - | - | - | - | T | T |
